# Enhanced Suppressive Activity of Regulatory T Cells in the Microenvironment of Malignant Pleural Effusions

**DOI:** 10.1155/2018/9876014

**Published:** 2018-03-26

**Authors:** Joanna Budna, Mariusz Kaczmarek, Agata Kolecka-Bednarczyk, Łukasz Spychalski, Piotr Zawierucha, Joanna Goździk-Spychalska, Michał Nowicki, Halina Batura-Gabryel, Jan Sikora

**Affiliations:** ^1^Department of Histology and Embryology, Poznan University of Medical Sciences, Poznan, Poland; ^2^Department of Clinical Immunology, Poznan University of Medical Sciences, Poznan, Poland; ^3^Department of Oncology, Poznan University of Medical Sciences, Poznan, Poland; ^4^Department of Anatomy, Poznan University of Medical Sciences, Poznan, Poland; ^5^Department of Pulmonology, Allergology and Respiratory Oncology, Poznan University of Medical Sciences, Poznan, Poland

## Abstract

Cancer metastatic spread to serous cavity causes malignant pleural effusions (MPEs), indicating dismal prognosis. Tumor microenvironment can implement suppressive activity on host immune responses. Thus, we investigated the prevalence of Tregs and the relationship between them and TGF-*β* and IL-10 concentrations and measured expression of *FOXP3*, *CTLA-4*, *CD28*, and *GITR* genes, as well as protein expression of selected genes in benign effusions and MPEs. The percentage of Tregs was determined by means of multicolor flow cytometry system. TGF-*β* and IL-10 concentrations were measured using human TGF-*β*1 and IL-10 ELISA kit. Relative mRNA expression of studied genes was analyzed by real-time PCR. The frequency of Tregs was significantly higher in MPEs compared to benign effusions; however, the level of TGF-*β* and IL-10 in analyzed groups was comparable, and no correlation between concentrations of TGF-*β* and IL-10 and percentage of Tregs was observed. Relative mRNA expression of all the genes was higher in CD4^+^CD25^+^ compared to CD4^+^CD25^−^ cells. In CD4^+^CD25^+^ cells from MPEs, relative mRNA expression of *FOXP3*, *CTLA-4*, and *CD28* genes was significantly higher than in benign effusions; however, the level of CD4^+^CD25^+^CTLA-4^+^ cells in analyzed groups showed no significant differences. We found numerous genes correlations in an entire CD4^+^CD25^+^ cell subset and CD4^+^CD25^+^ cells from MPEs. Enhanced suppressive activity of Tregs is observed in the microenvironment of MPEs. Understanding of relations between cellular and cytokine immunosuppressive factors in tumor microenvironment may determine success of anticancer response.

## 1. Introduction

Cancer metastatic spread to serous cavity often causes malignant pleural effusions (MPEs), indicates a dismal prognosis, and occurs in 15% of cancer-related deaths [1]. Severity of MPEs is related to the fact that contact between tumor-associated lymphocytes (TALs) and tumor cells is not hindered by connective tissues [2]. Prior studies have noted a strong relationship between tumor progression and T cell functional impairment, which can be explained by tumor's suppressive impact on host immune response [3].

Regulatory T cells (Tregs), a small subset (5–10%) of the overall CD4^+^ lymphocytes population, are defined by high expression of interleukin- (IL-) 2 receptor *α* chain (CD25), transcription factor FoxP3, a CTL-associated antigen-4 (CTLA-4), CD28, glucocorticoid-induced tumor necrosis factor (GITR), CD45RO, CD39, and CD73 [4]. Among them, FoxP3 seems to be the most relevant marker, since its presence and upregulated expression are required for Tregs development and function, preventing autoimmune diseases [5, 6]. However, its expression is not a unique feature of this subpopulation, since it can be found on CD4^+^CD25^−^ effector T cells [7], suppressor Tr1 and Th3 cells [8], or cancer cells [9].

It is widely known that Tregs, expressing FoxP3, are vital for self-tolerance, thus maintaining balance of immunological defense, by inhibiting effector T cells (Tef). This process occurs in two ways, by cell-to-cell direct contact or by secretion of inhibitory cytokines, like interleukin-10 (IL-10) and transforming growth factor-*β* (TGF-*β*) [10]. Both cytokines, acting as negative regulators, can lead to tumor progression [11, 12], since Tregs-mediated immunosuppression appears to be a crucial mechanism of tumor evasion, contributing to lack of response to immunotherapy in cancer patients [13]. Furthermore, it has been reported that Treg frequencies in patients with solid tumors and hematologic malignancies were higher than those in healthy controls [14]; however, the percentage of Tregs may vary between patients and type of cancer [15].

Tregs differentiate mainly in the thymus, but this process occurs also in the periphery. It is suggested that TGF-*β* is involved in this process, inducing differentiation of FoxP3^+^ Tregs from naive precursors [16]. In inflammatory effusions, pleural mesothelial cells play a key role in TGF-*β* synthesis, while in malignant effusions, TGF-*β* is produced mainly by tumor malignant cells [17].

Relationships between CTLA-4, CD28, and GITR receptors are considered to be responsible for Treg activity and suppressive function as they influence antigen-presenting cells (APC) stimulatory capacity [15]. Thus, the ability to control the suppressive function and/or number of Tregs in the cancer microenvironment has a promising therapeutic approach.

The present study investigates the prevalence of Tregs in malignant and benign pleural effusions, evaluates the relationship between them and TGF-*β* and IL-10 concentrations, and measures relative mRNA expression of *FOXP3*, *CTLA-4*, *CD28*, and *GITR* genes, as well as protein expression of selected genes.

## 2. Material and Methods

### 2.1. Patients

Pleural effusion samples, obtained by thoracocentesis from 76 patients admitted to the Greater Poland Centre of Pulmonology and Thoracic Surgery, were subjected to a routine laboratory diagnosis and analyzed by conventional cytology. Smears were fixed and stained with hematoxylin and eosin. Slides were evaluated as being negative or positive for malignant cells. Biological materials were divided into three groups: MPE with malignant cells (30, group I), effusions from patients with malignancy but without malignant cells in effusions (21, group II), and nonmalignant pleural effusions (25, group III). The last group consisted of tuberculosis and parapneumonic effusions.

In all patients, cytological diagnosis was confirmed by histology and clinical data. All malignant patients displayed effusions related to lung adenocarcinoma. None of the patients with MPE received any anticancer therapy. Among 51 patients with lung carcinoma, all were diagnosed as stage IV of the disease. The degree of advanced disease was established according to the 7th IASLC edition of TNM lung cancer classification.

Results of blood tests, including WBC, neutrophils, and monocytes counts, erythrocyte sedimentation rate (ESR), and CRP level, were collected for each patient.

### 2.2. Flow Cytometry Staining, Acquisition, and Analysis

The Treg levels (%) were measured in 76 PEs by flow cytometry with a use of Human Treg Flow™ Kit FoxP3 Alexa Fluor® 488/CD4 PE-Cy5/CD25 PE (BioLegend®, USA) according to the manufacturer's instructions. Briefly, cells were stained with combinations of the anti-CD4 PE-Cy5 and anti-CD25-PE antibodies. Samples were fixed and permeabilized. For intracellular staining of transcription factor FoxP3, Alexa Fluor 488 antihuman FoxP3 antibody or Alexa Fluor 488 mouse IgG1, k isotype control was used. Additionally, cells were stained with anti-CTLA-4-PerCP antibody. Data acquisition and analysis were performed on 5 × 10^4^ cells in samples using the FACSCanto™ II flow cytometry system and FACSDiva™ software (BD Biosciences) with a standard 6-color filter configuration. Lymphocytes were identified based on cell characteristic properties in the forward (FSC) and side (SSC) scatter and calculated based on staining with CD14PE/CD45FITC antibodies (Becton Dickinson, USA). For additional analyses, gates were restricted to the CD4^+^, CD4^+^CD25^+^, CD4^+^CD25^+^ FoxP3^+^, and CD4^+^CD25^+^CTLA-4^+^ cells.

### 2.3. Measurement of TGF-*β*1 and IL-10 Concentrations in Pleural Effusions

The TGF-*β*1 and IL-10 concentrations were measured in 76 samples with the use of enzyme-linked immunosorbent assay (ELISA) kit (Quantikine Human TGF-*β*1 and Quantikine Human IL-10, R&D Systems, USA). Before measurement, pleural effusions were centrifuged at 200 ×g for 10 min at 4°C. Supernatants were collected at kept in −80°C until ELISA was performed. The TGF-*β*1 and IL-10 concentrations were evaluated according to the manufacturer's instructions. Optical density of each well was determined within 30 min after blocking enzymatic reaction, using a microplate reader set to 450 nm with wavelength correction set to 540 nm. Measurements were carried out in duplicates, and results were calculated with a use of a standard curve.

### 2.4. Magnetic Isolation of CD4^+^CD25^+^ Regulatory T Cells from Pleural Effusions

The isolation of CD4^+^CD25^+^ regulatory T cells was performed on 33 samples with a use of CD4^+^CD25^+^ Regulatory T cell Isolation Kit human (MACS, Miltenyi Biotec, USA) and performed according to the manufacturer's instructions. Briefly, separation was carried out in a two-step procedure. First, the CD4^−^ cells were indirectly magnetically labeled with a cocktail of biotin-conjugated antibodies, and then antibiotin monoclonal antibodies conjugated to MicroBeads. Subsequently, labeled CD4^−^ cells were depleted on a column and placed in the magnetic field. In the second step, preenriched CD4^+^ cells were labeled with anti-CD25 antibody conjugated to MicroBeads, isolated by positive selection on a column, and placed in the magnetic field. The positive selection step was repeated twice to increase the purity of the fraction containing the CD4^+^CD25^+^ regulatory T cells. The unlabeled CD4^+^CD25^−^ cell effluent was also collected as a reference group.

### 2.5. RNA Isolation, Reverse Transcription, and Q-PCR Analysis

Total RNA was isolated from CD4^+^CD25^+^ white blood cells based on modified Chomczyński-Sacchi method using TRIzol® Reagent (Life Technologies, USA). Additionally, in order to purify probes, RNA was centrifuged on spin columns (Spin Cartridge and Collection Tube, Life Technologies, USA). The RNA samples were resuspended in 30 *μ*l of RNase-free water and stored in −70°C for further analysis. In the next step, isolated and purified RNA samples were used to carry out reverse transcription reaction using Transcriptor First Strand cDNA Synthesis Kit (Roche Diagnostics, Germany). To carry out Q-PCR, 5 *μ*l of reverse-transcribed cDNA was used in combination with 10 *μ*l Light Cycler 480® Probes Master Mix (Roche Diagnostics, Germany), 2 *μ*l primers (Table 1), and 0.2 *μ*M probes (Roche Diagnostics, Germany) with addition of 3 *μ*l H_2_O (Roche Diagnostics, Germany). Q-PCR was conducted in a Rotor Gene 6000 system (Corbett Research, Australia). To quantify changes in gene expression, the relative quantification method (2^−ΔΔCt^ method) has been used. GAPDH was used as housekeeping gene, and CD4^+^CD25^−^ cells were considered as the calibrator (reference) group. In order to eliminate technical issues, all samples were carried out in duplicates (technical repeats).

### 2.6. Statistical Analysis

The data were tabulated and analyzed using the STATISTICA 6.0 (StatSoft Inc., USA). To express the variability of data, standard deviation (SD) was used. In statistical analysis, we used nonparametric tests because of the nonnormal distribution of the data. The results were compared using the Mann–Whitney test to verify differences between two groups when 2 out of 3 analyzed groups were taken together, giving a total number of 2 compared groups, and the Kruskal-Wallis test with Dunn post hoc test to verify differences between more than two groups (cytometry analysis, ELISA test, and blood parameters, each consisting of 3 biological groups).

To analyze relative gene expression between two compared groups of pleural effusions, the Mann–Whitney *U* test was used.

Spearman's rank correlation coefficient and its significance were used to assess correlations between percentage of CD4^+^CD25^+^ regulatory T cells, TGF-*β* and IL-10 concentrations, and blood parameters, as well as correlation between analyzed gene expression changes.

## 3. Results

### 3.1. Flow Cytometry Analysis of Pleural Effusion Lymphocytes

**Figure 1:**
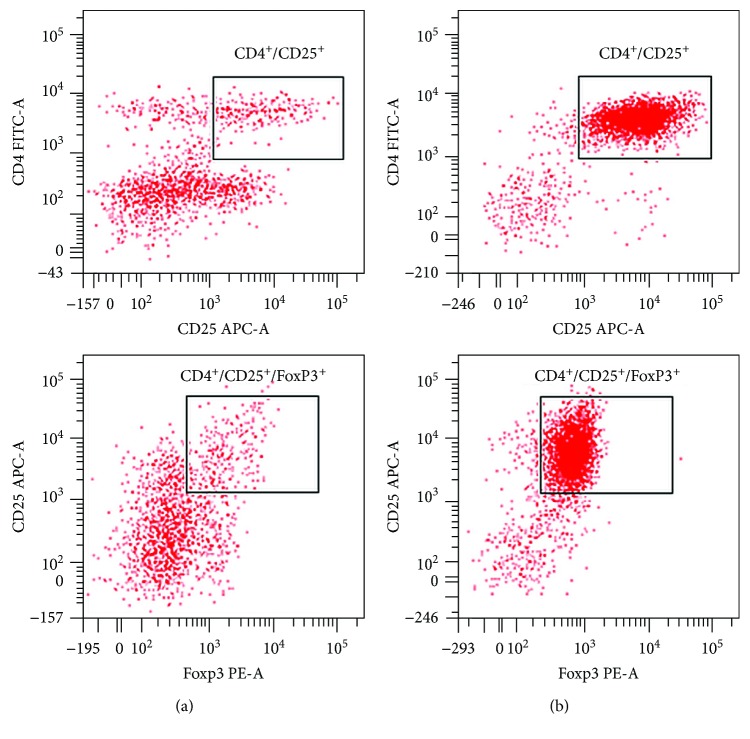
Scheme of immunophenotyping of the Tregs present in pleural effusions: (a) Tregs in pleural effusions; (b) Tregs in pleural effusions after magnetic separation of CD4^+^CD25^+^ cells.

Pleural effusions were analyzed by flow cytometry for phenotypic evidence of CD4^+^CD25^+^ FoxP3^+^ Tregs. After data acquisition, lymphoid cells were gated on FSC/SSC and analyzed for coexpression of CD4, CD25, and FoxP3 (Figure 1(a)). The final effect of CD4^+^CD25^+^FoxP3^+^ Treg enrichment by magnetic separation of CD4^+^CD25^+^ cells was evaluated using a flow cytometry (Figure 1(b)).

**Figure 2:**
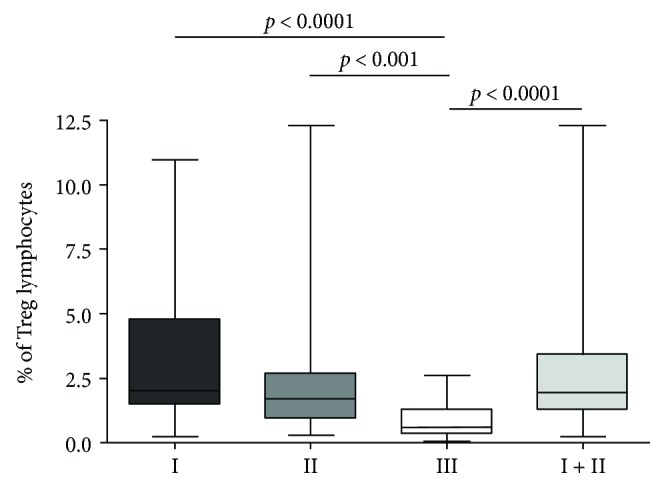
Frequencies of Tregs in three cytologically determined groups of pleural effusions: malignant effusions with malignant cells (I), malignant effusions without malignant cells (II), and nonmalignant pleural effusions (III).

We found statistically higher frequency of CD4^+^CD25^+^ FoxP3^+^ T cells in MPE with malignant cells (I) than in nonmalignant pleural effusions (III) (3.29% ± 2.99% versus 0.82% ± 0.66%, *p* = 0.000009) and in MPE without malignant cells (II) compared to nonmalignant pleural effusions (III) (2.46% ± 2.53% versus 0.82% ± 0.66%, *p* = 0.0015). The prevalence of CD4^+^CD25^+^FoxP3^+^ T cells in MPE containing malignant cells (I) and in MPE without malignant cells (II) was not significantly different (3.29% ± 2.99% versus 2.46% ± 2.53%, *p* > 0.05). Furthermore, the percentage of Tregs in both MPE taken together (I + II) was significantly higher than in nonmalignant pleural effusions (III) (2.92% ± 2.80% versus 0.82% ± 0.66%, *p* = 0.000002) (Figure 2). ^∗^Result already published in other aspects [18].

**Figure 3:**
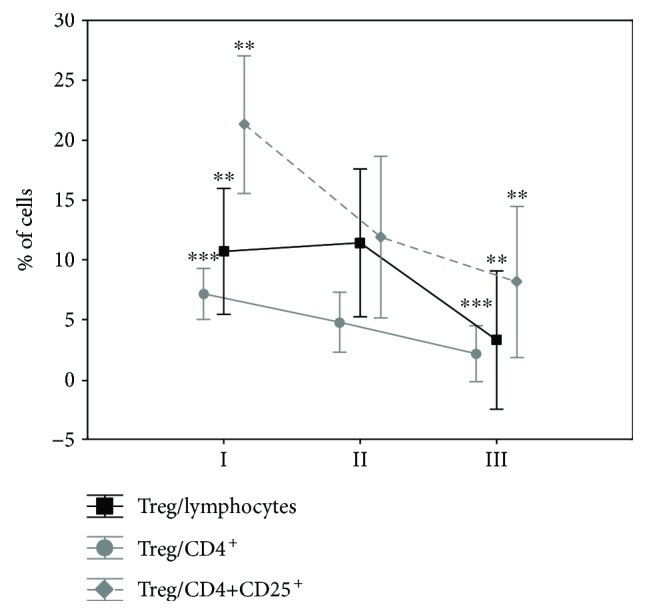
Frequencies of Tregs within all lymphocytes and CD4^+^ and CD4^+^CD25^+^ cells in three cytologically determined groups of pleural effusions: malignant effusions with malignant cells (I), malignant effusions without malignant cells (II), and nonmalignant pleural effusions (III). ^∗∗^*p* < 0.01; ^∗∗∗^*p* < 0.001

The frequency of lymphocytes in all effusion cells, and CD4^+^ lymphocytes and CD4^+^/CD25^+^ cells within lymphocytes, was evaluated. No significant differences were observed between these two populations. However, we found statistically significant differences in percentage of Tregs in lymphocytes between groups I and III (*p* = 0.006), in percentage of Tregs in CD4^+^ cells between groups I and III (*p* = 0.00018), and in percentage of Tregs in CD4^+^CD25^+^ cells between groups I and III (*p* = 0.004) (Figure 3).

**Figure 4:**
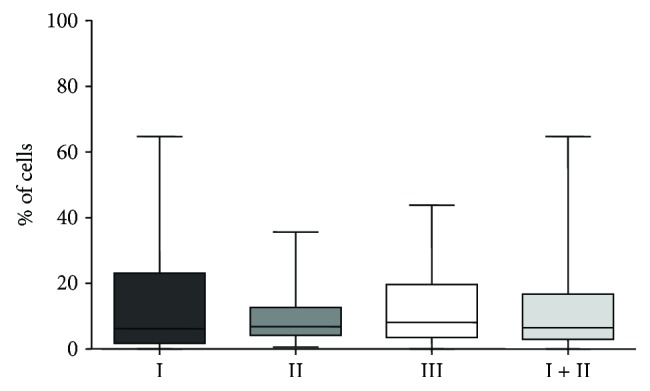
Frequencies of CD4^+^CD25^+^CTLA-4^+^ cells in three cytologically determined groups of pleural effusions: malignant effusions with malignant cells (I), malignant effusions without malignant cells (II), and nonmalignant pleural effusions (III).

There was no statistically significant difference between percentage of CD4^+^CD25^+^CTLA-4^+^ cells in any of analyzed groups (I versus III, *p* = 0.5640; II versus III, *p* = 0.6643, I + II versus III, *p* = 0.5468; and I versus II, *p* = 0.8395) (Figure 4).

### 3.2. TGF-*β*1 and IL-10 Concentrations in Pleural Effusions

**Figure 5:**
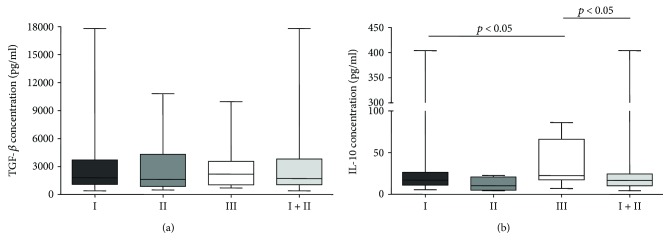
TGF-*β*1 (a) and IL-10 (b) concentrations in three cytologically determined groups of pleural effusions: malignant effusions with malignant cells (I), malignant effusions without malignant cells (II), and nonmalignant pleural effusions (III).

Average TGF-*β*1 concentrations in MPE with malignant cells were 2857 pg/ml, in MPE without malignant cells 2999 pg/ml, and in benign pleural effusions 2745 pg/ml. Statistical analysis showed no significant differences between MPE with and without malignant cells and benign pleural effusions (*p* = 1.00), between both malignant groups (*p* = 1.00), and between both MPE taken together and benign effusions (*p* = 0.9702). Results are presented in Figure 5(a).

Average IL-10 concentrations in MPE with malignant cells were 35 pg/ml, in MPE without malignant cells 23 pg/ml, and in benign pleural effusions 39 pg/ml. Similarly, statistical analysis showed no significant differences between both malignant groups (*p* = 0.6908), and group of MPE without malignant cells and benign effusions (*p* = 0.0903). However, between MPE with malignant cells and benign pleural effusions (*p* = 0.0286), and between both MPE taken together and benign effusions (*p* = 0.0279), differences were statistically significant. Results are presented in Figure 5(b).

### 3.3. Correlation between Frequency of Tregs and TGF-*β*1 and IL-10 Concentrations in Pleural Effusions

**Figure 6:**
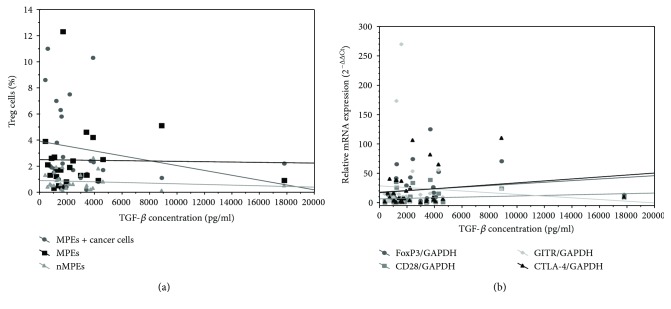
Correlation between TGF-*β*1 concentration and (a) frequency of Tregs in MPEs-malignant pleural effusions with malignant cells, MPEs-malignant pleural effusions without malignant cells, and nMPEs-nonmalignant pleural effusions and (b) relative mRNA expression of *FOXP3*, *CD28*, *CTLA-4*, and *GITR* genes in CD4^+^CD25^+^ cells isolated from all pleural effusions.

Statistical analysis (Spearman correlation coefficient) showed no correlation between frequency of Tregs and concentration of TGF-*β*1 in any group of pleural effusions separately (MPEs with malignant cells: *p* = 0.105; MPEs without malignant cells: *p* = 0.0598; nMPEs: *p* = 0.369) (Figure 6(a)).

Similarly, there was no significance in correlation between frequency of Tregs and concentration of IL-10 in any group of pleural effusions separately (MPEs with malignant cells: *p* = 0.227; MPEs without malignant cells: *p* = 0.193; nMPEs: *p* = 0.364). Among tested groups, only MPEs without malignant cells presented a positive correlation trend.

### 3.4. Relationship between Frequency of Tregs and/or TGF-*β*1 Concentrations and Concomitant Immune Activation in Pleural Effusions

Statistical analysis (Spearman correlation coefficient) showed no correlation between frequency of Tregs and potential immune activation reflected by WBC, neutrophils, and monocytes counts, ESR, and CRP level, within tested groups of effusions. However, we found a statistically significant correlation between TGF-*β*1 concentration versus monocytes count and TGF-*β* versus ESR in MPEs with malignant cells (*r*_*s*_ = 0.69 and *p* = 0.006; *r*_*s*_ = 0.53 and *p* = 0.049, resp.), and in nMPEs (*r*_*s*_ = 0.67 and *p* = 0.045; *r*_*s*_ = 0.87 and *p* = 0.0009, resp.).

Comparing factors reflecting immune activation between groups, we found statistically significant differences in monocytes count between MPEs with malignant cells and nMPEs (*p* = 0.0072), and in ESR between MPEs with malignant cells and MPEs without malignant cells, as well as between the latter group and nMPEs (*p* = 0.037 for both). Furthermore, we found statistically significant differences in WBC, neutrophils, and monocytes counts between both MPEs taken together (I + II) and nMPEs (III) (*p* = 0.022; *p* = 0.037; *p* = 0.0055, resp.).

### 3.5. Relative mRNA Expression of *FOXP3*, *CD28*, *CTLA-4*, and *GITR* Genes in CD4^+^CD25^+^ Cells from Pleural Effusions

**Figure 7:**
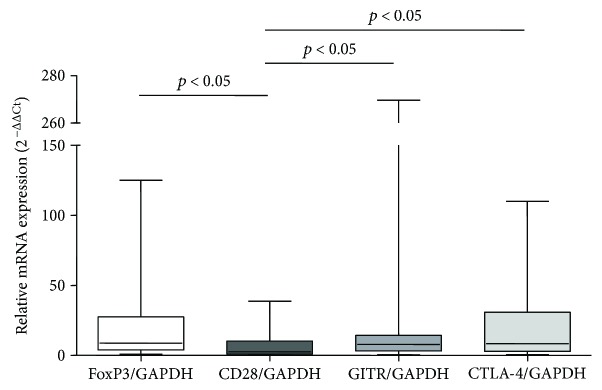
Relative mRNA expression of *FOXP3*, *CD28*, *GITR*, and *CTLA-4* genes in CD4^+^CD25^+^ cells in relation to CD4^+^CD25^−^ cells from all pleural effusions.

The level of expression was evaluated in CD4^+^CD25^+^ cells in relation to CD4^+^CD25^−^ cells isolated from pleural effusions. We observed increased expression of all four genes in CD4^+^CD25^+^ cells in comparison to expression in CD4^+^CD25^−^ cells, which was taken as 1. The level of *FOXP3* expression increased 21-fold (median 9-fold), *CTLA-4* 21-fold (median 8-fold), *GITR* 23-fold (median 8-fold), and *CD28* 8-fold (median 3-fold) (Figure 7).

**Figure 8:**
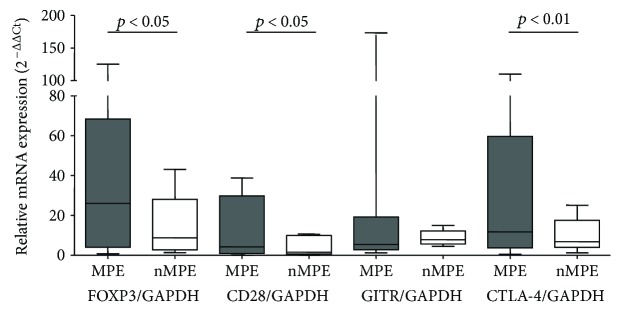
Relative mRNA expression of *FOXP3*, *CTLA-4*, *CD28*, and *GITR* genes in CD4^+^CD25^+^ cells in relation to CD4^+^CD25^−^ cells from malignant and benign pleural effusions.

The relative mRNA expression of all four genes in CD4^+^CD25^+^ cells in relation to CD4^+^CD25^−^ cells was compared between MPE with malignant cells and benign pleural effusions. For *FOXP3* gene, average was 30 (median 15) in MPE with malignant cells and 9 (median 7) in benign pleural effusions; for *CTLA-4* gene, 32 (median 19) and 7 (median 5), respectively; for *CD28* gene, 12 (median 4) and 2 (median 1), respectively; and for *GITR* gene, 35 (median 9) and 7 (median 7), respectively. A statistically significant increase was observed in MPE with malignant cells in comparison to benign effusions for *FOXP3* (*p* = 0.047), *CTLA-4* (*p* = 0.009), and *CD28* (*p* = 0.017) genes. There was no increase observed for *GITR* gene (*p* = 0.43) as far as median values were concerned; however, average values were considerably different. Lack of statistically significant differences was caused by great dispersion of results (1–270 in MPEs with malignant cells and 0.5–16 in benign effusions) (Figure 8).

### 3.6. Correlations between Relative mRNA Expression of *FOXP3*, *CD28*, *CTLA-4*, and *GITR* Genes in CD4^+^CD25^+^ Cells Isolated from Pleural Effusions and TGF-*β* Concentration

Statistical analysis (Spearman correlation coefficient) showed no correlation between relative mRNA expression of *FOXP3*, *CD28*, *CTLA-4*, and *GITR* genes and concentration of TGF-*β*1 in all pleural effusions (*p* = 0.13, *p* = 0.32, *p* = 0.50, and *p* = 0.88, resp.) (Figure 6(b)).

### 3.7. Correlations between Relative Expression Levels of *FOXP3*, *CD28*, *CTLA-4*, and *GITR* Genes in CD4^+^CD25^+^ Cells Isolated from Pleural Effusions

The correlation analysis included CD4^+^CD25^+^ cells from all collected pleural effusions. Results indicate that among all genes, only *CD28* and *GITR* genes' relative expression levels did not correlate. The rest was statistically significant (Table 2, PEs column).

A subsequent correlation analysis included CD4^+^CD25^+^ cells from malignant pleural effusions with malignant cells. Similar to previous results, only *CD28* and *GITR* genes' relative expression levels did not correlate. The rest was statistically significant (Table 2, MPEs column).

The last correlation analysis included CD4^+^CD25^+^ cells from nonmalignant pleural effusions. We observed a statistically significant correlation between *FOXP3* and *CD28*, *FOXP3* and *CTLA-4*, and *CD28* and *CTLA-4* genes' expression levels, whereas *FOXP3* and *GITR*, *CD28* and *GITR*, and *CTLA-4* and *GITR* genes' relative expression levels did not correlate (Table 2, nMPEs column).

## 4. Discussion

Studies conducted on MPEs enable one to better understand the mechanisms governing human immune response in tumor microenvironment. Since the majority of cancers are characterized by increased frequency of Tregs, it is assumed that selective in vivo elimination of these cells would enhance antitumor response. Some attempts have already been made in studies with animal models, where addition of anti-CD25 monoclonal antibodies significantly increased antitumor response [19].

Increased Treg frequency in tumor-infiltrating lymphocytes (TILs) was first shown in ovarian and non–small cell lung cancer (NSCLC) by Woo et al. [20]. Many other authors reported similar findings both in peripheral blood of cancer patients and in tumor microenvironment [21–24]. Interestingly, no differences in Treg frequencies among squamous cell carcinoma and adenocarcinoma were found, which suggested that the mechanism triggering Treg expansion is cancer histological type-independent [25]. On the other hand, Treg counts differ among cancer types. DeLong et al. found a significantly higher percentage of functional CD4^+^CD25^+^ cells in PEs caused by NSCLC and breast cancer, compared to mesothelioma [26].

Similarly, we found a statistically significant increase in CD4^+^CD25^+^FoxP3^+^ frequency in MPEs over the course of lung cancer, with the highest Treg percentage in MPEs with malignant cells. Furthermore, we observed higher percentage of Tregs within all lymphocytes, and CD4^+^ and CD4^+^CD25^+^ lymphocytes in MPEs with malignant cells, compared to benign effusions. This observation suggests that malignant cells play a substantial role in enhanced induction, proliferation, and/or migration of CD4^+^CD25^+^FoxP3^+^ cells from the periphery to tumor microenvironment. Especially higher percentage of Tregs within activated Tef in MPEs with malignant cells than in benign effusions confirms this hypothesis. Moreover, our previous studies correlating Treg frequency with patient survival showed that patients with lower percentage of Tregs lived longer did than those with higher Treg incidence; however, this finding was not statistically significant [18].

Among T cells present in MPE, Tregs comprise up to 30% [27]. It was shown that elevated Treg counts are caused by de novo proliferation instead of migration from secondary lymphoid organs [28]. Additionally, tumor microenvironment, being also inflammatory, contains substantial amount of TGF-*β* cytokine associated with ability to convert CD4^+^CD25^−^ into CD4^+^CD25^+^FoxP3^+^ cells. Importantly, induced Tregs (iTregs) presented full suppressive activity resembling nTregs that has arisen in the thymus, demonstrated by diminished proliferation and IFN-*γ* production by CD4^+^CD25^−^ cells [29].

Atanackovic et al. found a 5-fold higher TGF-*β* level in MPEs in patients suffering from breast, esophageal, and pancreatic cancer and sarcoma compared to nonmalignant PEs. Furthermore, a higher TGF-*β* concentration in tumor microenvironment was related to higher frequency of CD4^+^CD25^+^FoxP3^+^ cells, compared to peripheral blood of the same individual [30]. Previously, Sikora et al. analyzed malignant and benign PEs and showed significantly increased concentration of TGF-*β* in the first group [31].

Contrary to expectations, this study did not find significant differences between groups of malignant (with or without malignant cells) and benign PEs. Moreover, no correlations were observed between frequency of CD4^+^CD25^+^FoxP3^+^ and TGF-*β* concentration in particular groups. It remained in accordance with observation made by other groups [32, 33]. However, we cannot exclude the possibility that the level of active TGF-*β* in vivo correlates with Treg incidence. This inconsistency may be due to the fact that according to the ELISA procedure, latent TGF-*β* (LAP and LTBP complexes) is activated, and as a result, we obtain total TGF-*β* concentrations, including primarily inactive, which does not take part in cell conversion in vivo. Moreover, apart from free form, Tregs can present membrane-bound TGF-*β*, which can effectively mediate a subsequent Treg conversion. Finally, in some types of cancer, like small cell lung cancer (SCLC), tumor cells do not produce TGF-*β*, which was shown by the lack of mRNA in these cells [34]. In this case, high and comparable levels of TGF-*β* can be caused by macrophages, abundantly present in all types of effusions [35].

Our present study supported indirectly this theory, since we correlated increase of TGF-*β* concentration and precursors of macrophages-blood monocytes in MPEs with malignant cells and nMPEs. Moreover, ESR, known as a marker of immune activation, also correlated with anti-inflammatory TGF-*β*. We assume this phenomenon can be explained by the starting phase of negative feedback loop, when advanced inflammation increases concentration of TGF-*β*, essential for following immune reaction attenuation. It clearly indicates that inflammation present in the microenvironment of PEs even has a systemic range.

The above theory can be also implied to clarify our observation of surprisingly a higher IL-10 level in nMPEs. Previous findings showed significantly higher concentration of IL-10 in MPEs, compared to nMPEs [36], as well as no differences between groups [37–39]. A comparable, or even higher, level of IL-10 in nMPEs can be also caused by the fact that pathogens, including *Mycobacterium tuberculosis*, supposedly present in our benign effusions, selectively upregulate IL-10 production. This mechanism, exploiting immunosuppressive properties of IL-10, creates a more advantageous environment for pathogens' survival. The mentioned *Mycobacterium tuberculosis* was found to regulate TLR4-mediated LPS signaling, resulting in downregulation of TNF*α* expression but profuse IL-10 production [40]. Alternative mechanism included modulation of TLR2 signaling, enhancement of IL-10-producing abilities of myeloid cells (i.e., abundant in nMPEs macrophages), and the induction of IL-10-secreting Tregs, thus impairing antimicrobial control [41]. Importantly, in the group of malignant PEs, with lower IL-10 level, we found a trend towards correlation between Treg percentage and IL-10 concentration, which is in agreement with general knowledge and proves that activated Tregs produce IL-10.

Although factors triggering and governing inflammation in cancerous and noncancerous diseases are considerably different, substantial immune activation appears in both conditions [42]. We observed that blood parameters, especially monocytes, and ESR significantly differed between PEs groups. Higher values of all parameters in malignant groups (separately and taken together) compared to the nonmalignant group showed that higher Treg frequency was convergent with concomitant immune activation. Increased values, reflecting chronic inflammation in cancer, seem to favor Treg accumulation in PE, possibly via previously mentioned TGF-*β* abundance.

Since all our pleural effusions contained TGF-*β*, we can assume that a great part of Tregs was induced (iTregs). It was shown that the Treg activation mechanism triggered by TGF-*β* includes epigenetics, that is, demethylation of CpG islands in the first intron of *FOXP3* gene, and participation of TGF-*β*-activated transcription factor Smad3 in *FOXP3* gene expression, supporting the idea of TGF-*β*-dependent *FOXP3* expression in iTregs [43].

In this study, *FOXP3* expression was significantly upregulated in CD4^+^CD25^+^ group compared to CD4^+^CD25^−^, which lets us assume that CD4^+^CD25^+^ cells were predominantly Tregs [44]. Moreover, CD4^+^CD25^+^ cells isolated from MPEs expressed *FOXP3* substantially stronger than did those from benign effusions, which was confirmed by protein level measured by flow cytometry. It can imply that both higher expression of FoxP3 protein and stronger inhibitory activity of these cells in tumor microenvironment, since upregulated expression of FoxP3, correlated with decreased level of proinflammatory cytokines, like tumor necrosis factor (TNF), IL-2, or granulocyte-macrophage colony-stimulating factor (GM-CSF), and on the other hand with increased expression of immunosuppressive cytokines, like IL-10 or TGF-*β* [45]. Nevertheless, a higher *FOXP3* level can also be an effect of higher frequency of FoxP3^+^ cells in CD4^+^CD25^+^ subpopulation in MPEs, which we determined previously. Additionally, some reports suggest that Tregs can lose FoxP3 expression triggered by proinflammatory cytokines, that is, IL-6 [46]. Since nonmalignant PEs were mainly inflammatory, it can explain decreased expression of *FOXP3* gene in this group.

Correlation between upregulated *FOXP3* expression, cancer stage, shorten progression-free survival, and poor prognosis was observed in breast [47], ovarian [48], and stomach cancers [49]. In addition, analysis of Treg frequency and *FOXP3* mRNA level in patients with esophageal cancer showed a decrease of both factors after chemotherapy, suggesting that FoxP3 inhibition can be an effective strategy in cancer therapy [50].

Although CTLA-4 expression is restricted only to activated Tef, it is constitutively expressed in Tregs. As the result of this study, we found significantly higher *CTLA-4* expression in CD4^+^CD25^+^ cells than in CD4^+^CD25^−^ cells, which correlated with *FOXP3* increased expression. Similarly, Zheng et al. found the same relation between both proteins [51], who suggested that FoxP3 together with other transcription factors, that is, NFAT, can regulate transcription of *CTLA-4* gene; however, cells transfected with *CTLA-4* not always presented FoxP3 expression [52]. Thus, the role of FoxP3 in CTLA-4 expression is still controversial, especially that the latest data indicate that on the contrary, CTLA-4 may be responsible for FoxP3^+^ Treg occurrence [53].

Furthermore, we observed considerably higher *CTLA-4* expression in CD4^+^CD25^+^ cells derived from MPEs than in benign effusions. So far, there is no information about this phenomenon in lung cancer; however, there exist some data from breast and colon cancer, which are consistent with ours. Jaberipour et al. found an increased level of both *FOXP3* and *CTLA-4* gene transcript and correlation between them in PBMC of breast cancer patients compared to a control group of healthy women. The high level of transcripts in the early stages of the disease implies that Tregs play a key role in cancer expansion from the beginning [54]. *CTLA-4* expression was associated also with breast cancer stage [55] and was confirmed by protein expression in cervical cancer [56]. Unfortunately, we were unable to show differences in CTLA-4 protein level measured by flow cytometry. Conversely, Lee et al. observed decreased CTLA-4 expression in patients suffering from colon cancer in all stages of the disease in comparison to that in the control group [57].

Unlike CTLA-4, CD28 expression in Tef is not restricted to activated cells. Thus, common expression of CD28 in both effector and regulatory T cells can explain smaller differences in transcript level between CD4^+^CD25^+^ and CD4^+^CD25^−^ cells observed in our study. Nevertheless, differences were significant and could be caused by the presence of Tregs from MPEs, with functions enhanced by higher CD28 expression. Moreover, there was a clear relation between *CD28* and *CTLA-4* gene increase, indicating that in Tregs from PEs, the level of activation is associated with improved suppression ability.

Studies including whole population of peripheral or tumor-infiltrating T cells showed that in NSCLC [58] and breast [59], cervical [56], and colon cancers [57], CD28 was downregulated while apoptotic receptor CD95 was upregulated. All these indicate that CD28 along with CD95 plays an essential role in lung cancer progression; however, decrease in CD28 expression was possibly the result of activation loss among Tef (especially CD3^+^ and CD8^+^), not Tregs, which especially in elderly people can lead to impaired immune response and tumor spread. We found a very substantial difference in *CD28* expression between CD4^+^CD25^+^ cells isolated from malignant versus benign PEs. Although our results of higher *CD28* expression in cancerous compared to benign group of effusions differ from those mentioned above, a possible explanation for this might be the higher Treg activation in tumor microenvironment. In contrast to the malignant group, in the benign group, the same correlation was weaker, which may be explained by Tregs' lower activation or lower frequency among CD4^+^CD25^+^ cells.

Interestingly, Lee et al. observed a significantly higher *CD28* mRNA level compared to *CTLA-4* in patients with lymph node metastasis versus metastasis-free group in colon cancer, which, along with immunohistochemical analysis, might be a promising diagnostic tool for determining cancer progression [57].

It is known that GITR is present on Treg surface as well as to some extent on naive T cells, where it is upregulated after activation [60]. Our studies support this theory, since *GITR* mRNA level was much higher in CD4^+^CD25^+^ cells compared to CD4^+^CD25^−^ cells. Furthermore, we found a strong correlation between increase of *GITR*, *FOXP3*, and *CTLA-4* transcripts, pointing to the considerable Treg contribution in the CD4^+^CD25^+^ subset. Similar to *CTLA-4*, the presence of *GITR* in CD4^+^CD25^−^ subset can be related to its activation state.

We observed higher *GITR* expression in CD4^+^CD25^+^ cells recruited from MPEs compared to benign; however, the difference was not significant. Additionally, in the malignant group, correlations between *GITR*, *FOXP3*, and *CTLA-4* transcripts were parallel to those in total CD4^+^CD25^+^ subpopulation, suggesting substantial share of cells with FoxP3, CTLA-4, and GITR coexpression in cells from the malignant cohort.

Baltz et al. noticed that GITR-L is present on many cancer cell lines, including lung cancer. Thus, Tregs and malignant cells coincidence in tumor microenvironment would favor the Treg-suppressive activity. Moreover, GITR-L presence in cancer cells was associated with strengthened TGF-*β* production, also supporting Treg expansion [61].

## 5. Conclusions

We observed enhanced suppressive activity of Tregs in the microenvironment of MPEs. Understanding the relations between cellular and cytokine immunosuppressive factors provides new insight into mechanisms of Treg activation in tumor microenvironment and their role in anticancer response. A key to successful anticancer immunotherapy is selective abrogation of tumor immunotolerance, while maintaining tolerance for host antigens. Thus, not only Treg elimination but also blocking/activation of their receptors, and immunosuppressive cytokine deprivation, can be implemented in a variety of therapies.

## Figures and Tables

**Table 1:** Oligonucleotide sequences used for Q-PCR analysis.

Transcript	Sequence (5′-3′ direction)		Gene accession no.	Product size
FoxP3	F	AGGAGGATGGACGAACAGG	NM_014009.3	76 bp
	R	CACATCCAGGGCCTATCATC		
CD28	F	CATGGCCCAAGTCTGTCTTT	NM_006139.3	63 bp
	R	TGTATGTCGGGCATGCTACT		
CTLA-4	F	CCGTGCCCAGATTCTGAC	NM_005214.4	60 bp
	R	AAACAACCCCGAACTAACTGC		
GITR	F	GACCGAAGACGCCAGAAG	NM_004195.2	95 bp
	R	CTCACACCCACAGGTCTCC		
GAPDH	F	GCATCCTGGGCTACACTGA	NM_002046.5	79 bp
	R	CCAGCGTCAAAGGTGGAG		

**Table 2:** Correlations between analyzed genes in three groups of pleural effusions.

	PEs	MPEs	nMPEs
*FOXP3*/*CD28*	*r_s_* = 0.794452 *p* < 0.0001	*r_s_* = 0.828070 *p* < 0.0001	*r_s_* = 0.652747 *p* < 0.05
*FOXP3*/*GITR*	*r_s_* = 0.386698 *p* < 0.05	*r_s_* = 494737 *p* < 0.05	*r_s_* = 0.116484 *p* = 0.6916
*FOXP3*/*CTLA-4*	*r_s_* = 0.749332 *p* < 0.0001	*r_s_* = 0.743860 *p* < 0.001	*r_s_* = 0.665934 *p* < 0.05
*CD28*/*GITR*	*r_s_* = 0.233957 *p* = 0.19	*r_s_* = 0.412281 *p* = 0.079	*r_s_* = −0.468132 *p* = 0.0913
*CD28*/*CTLA-4*	*r_s_* = 0.892714 *p* < 0.0001	*r_s_* = 0.868421 *p* < 0.0001	*r_s_* = 0.617582 *p* < 0.05
*GITR*/*CTLA-4*	*r_s_* = 0.495655 *p* < 0.05	*r_s_* = 0.650877 *p* < 0.05	*r_s_* = 0.178022 *p* = 0.5425

Correlations between relative mRNA expression of *FOXP3*, *CD28*, *CTLA-4*, and *GITR* genes in CD4^+^CD25^+^ cells isolated from: PEs = all pleural effusions; MPEs = malignant pleural effusions with malignant cells; and nMPEs = nonmalignant pleural effusions.
